# Tooth Discoloration Resulting from a Nano Zinc Oxide-Eugenol Sealer

**DOI:** 10.22037/iej.2017.15

**Published:** 2017

**Authors:** Mina Zarei, Maryam Javidi, Marzieh Jafari, Maryam Gharechahi, Pedram Javidi, Maryam Shayani Rad

**Affiliations:** a *Dental Research Center, Department of Endodontics, Dental School, Mashhad University of Medical Science, Mashhad, Iran; *; b * Dental Research Center, Department of Endodontics, Dental School, Mashhad University of Medical Science, Mashhad, Iran; *; c * Dental school, North Khorasan University of Medical Science , Bojnurd, Iran; *; d * Dental Materials Research Center, Department of Endodontics, Dental School, Mashhad University of Medical Science, Mashhad, Iran; *; e *Department of Orthodontics, Dental School, Ahvaz Jundishapur University of Medical Sciences, Ahvaz, Iran; *; f *Student Research Committee (SRC), Mashhad University of Medical Sciences, Mashhad, Iran*

**Keywords:** Nano Particle, Root Canal Sealer, Spectrophotometry, Tooth Discoloration, Zinc-Oxide Eugenol

## Abstract

**Introduction:**

A desirable quality of any endodontic sealer is its ability to be tooth color friendly. Therefore the aim of the present study was to evaluate the tooth discoloration potential of a nano zinc oxide-eugenol (NZOE) sealer.

**Methods and Materials:**

In order to evaluate tooth discoloration, the pulp chamber of 60 human maxillary central and lateral incisors were filled with one of the sealers, naming AH-26 (resin-based sealer), Pulpdent sealer (ZOE-based) and a NZOE experimental sealer. Color measurements was assessed at the baseline (before placement of sealers) (T0), 24 h (T1) and 72 h (T2) h, 1-week (T3), and 1-month (T4) after the placement of sealers using the Easy Shade spectrophotometer. Data were analyzed in SPSS software using one-way ANOVA, and repeated measured ANOVA.

**Results:**

No significant differences were observed when the paired comparison test was performed (*P*>0.05).

**Conclusion:**

The tested NZOE sealer had similar tooth discoloration potential in comparison with AH-26 and ZOE sealer.

## Introduction

Sealing the root canal system after cleaning and shaping, is one of the essentials of endodontic treatment [1]. The presence of sealer is necessary regardless of the type of core material because it creates a strong fluid-tight seal [2]. Besides functional and biological characteristics, one of the principal properties of a sealer is its ability to prevent tooth discoloration [3]. The discoloration caused by endodontic materials including sealers used in endodontic treatment, is a common clinical problem [4]. Some of the main reasons for tooth discoloration are the presence of blood, necrotic tissues and endodontic materials. Studies evaluating the effect of root canal sealers on tooth crown discoloration have concluded that all the sealers used in endodontics have discoloration potential to some extent [3, 5].

The most commonly used sealers in root canal treatment are zinc-oxide eugenol (ZOE)-based sealers. Studies have shown that these sealers have a long history of successful use because of many positive properties such as profound antimicrobial activity [3]; however, one of the disadvantages of ZOE sealers, is tooth discoloration potential [6, 7]. On the other hand, resin-based sealers offer the advantage of adhering to the root canal walls and being free of eugenol. Many studies conducted on discoloration potential of sealers have insisted on the high discoloration potential of resin sealers [3]. It has been shown that AH-26 causes severe discoloration of tooth structures, which is attributed to the silver ions in its composition [8].

Recently, nanotechnology is extensively used in manufacturing dental materials [9]. The chief aim in this context is to prepare materials with better mechanical properties, lower resistance to abrasion and more favorable optical and esthetic properties [10]. Some other advantages of nanoparticles which have drawn attention in endodontics are penetration into dentinal tubules, antibacterial activity and low microleakage property [10, 11]. 

A new endodontic sealer with nano-sized ZOE powder particles (NZOE) has been developed in the Dental Material Research Center, Mashhad University of Medical Sciences, Mashhad, Iran. This sealer is similar to various ZOE-based sealers, but with different sizes of ZOE nanoparticles [12]. This sealer exhibit cytocompatibility [13] and antibacterial activity [11] with satisfactory sealing ability [12]. 

Tooth color assessment can be performed by visual color evaluation with shade guides [14], determination of dentine color changes in longitudinal tooth sections [15], digital imaging [16] and colorimeters and spectrophotometers [17]. Spectrophotometers is the reference method because of its high sensitivity, repeatability and data stability [18].

The aim of the present *in vitro* study, was to compare the tooth discoloration caused by NZOE, to two common endodontic sealers AH-26 (resin-based sealer), Pulpdent sealer (ZOE-based).

## Materials and Methods

**Figure 1:**
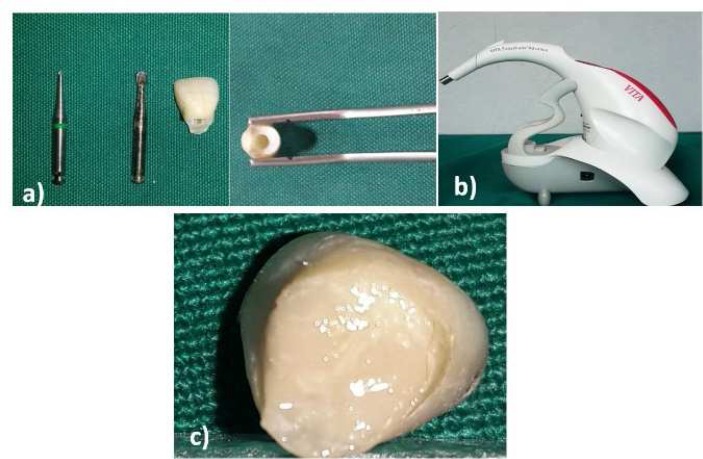
*A)* Tissue and pulp horn removal with a bur; *B)* Easy Shade Colorimetric device; *C)* sealing the access with glass-ionomer

The research protocol was approved by the Vice Chancellor of Research of Mashhad University of Medical Sciences (Grant No.: 920816). Sixty sound extracted maxillary central and lateral incisors, without any caries, cracks, fractures or restorations, were selected. First the tooth surfaces were polished to remove extrinsic discoloration. Then the tooth roots were separated from the crowns at cervical thirds of the roots. The pulp tissue and the pulp horns, if any, were removed by #4 and then 1/4 round burs (Brasseler USA, Savannah, GA, USA) installed on a slow-speed handpiece (Figure 1A). The canals were irrigated with sodium hypochlorite and normal saline at the end. The samples were randomly divided into three groups (*n*=20) according to the experimental sealers: Pulp Canal Sealer (SybronEndo, Orange, CA, USA), AH-26 sealer (Dentsply, De Trey, Konstanz, Germany) and NZOE. At the beginning of the procedures, the tooth color was determined with the use of Spectrophotometer (VITA Easyshade; VITA Zahnfabrik, Bäd Sackingen, Germany) (Figure 1B) twice and the mean value was recorded as the baseline value. 

Each sealer was mixed properly based on the standard protocol. AH-26 sealer was mixed at a powder-to-liquid ratio of 1:3 to achieve a homogeneous mix. A 1:1 powder-to-liquid ratio was used for pulp canal and NZOE sealers with adequate mixing. Each sealer was placed into the pulp chamber *via* the cervical access and was properly sealed with self-cure glass-ionomer (Fuji I LC, GC Corporation, Tokyo, Japan) (Figure 1C). The samples were incubated at 37^°^C under saturated moisture. Also the specimens were preserved in humid environment during color assessment in order to simulate the clinical situation considering that tooth dehydration may affect its optical properties reversibly [19].

***Color measurement***

The chromatic alterations were assessed by an intraoral Spectrophotometer (VITA Easyshade; VITA Zahnfabrik, Bäd Sackingen, Germany). All the measurements were carried out between 9 and 11 in the morning under identical light conditions in similar environment to minimize the environmental factors effect.

Color measurement was performed at the baseline (before placement of sealers) (T_0_), 24 h (T_1_) and 72 h (T_2_), 1-week (T_3_), and 1-month (T_4_) after the placement of sealers. The measurements were repeated twice for each specimen and the mean CIE parameters including* L *(the degree of darkness and lightness related to value in the Mansell color system), *a *(color coefficients related to hue in the Mansell color system), *b* (color coefficients related chroma in the Mansell color system) were recorded. In the color environment, based on this measurement the parameter depends on the color intensity on the green and red axes and the *b* parameter depends on the color intensity on the blue and yellow axes [4]. 

Color changes (∆E) for each time intervals were calculated with the following formula:

$∆E=\sqrt[{2}]{({a_{1}-a_{2})}^{2}+{\left(b_{1}-b_{2}\right)}^{2}+{\left(L_{1}-L_{2}\right)}^{2}}$

*ΔE*_1__=_colour difference between T_0_ and T_1_

*ΔE*_2__=_colour difference between T_0_ and T_2_

*ΔE*_3__=_colour difference between T_0_ and T_3_

*ΔE*_4__=_colour difference between T_0_ and T_4_

Standard threshold of *ΔE* is 3.7 (*ΔE*<3.7) (17). Data were analyzed with SPSS software version 16 (SPSS version 16, Chicago, IL, USA). The one-way ANOVA and repeated measured ANOVA tests were used for statistical analyses.

## Results

**Figure 2:**
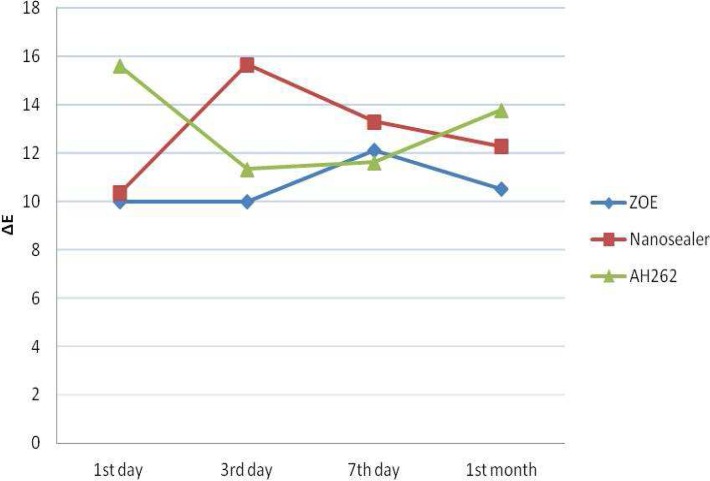
Tooth color change during 1 month

Table 1 presents the means ∆E and standard deviations (SD) of tooth discoloration values of each sealer at different time intervals. Discoloration caused by all three sealers in all measurement points was more than standard threshold (*ΔE*>3.7) but with no significant difference compared one another (*P*=0.112). The severity of discoloration at T_1_ and T_4_ was: AH-26>NZOE> Pulp Canal Sealer. This sequence altered to NZOE>AH-26> Pulp Canal Sealer at T_2_. At T_3_ the discoloration was as follows: NZOE> Pulp Canal Sealer>AH-26. After 3 days (until the 30^th^ day; the end of the experiment), tooth discoloration was progressive for AH-26, but tends to decrease for NZOE (Figure 2) but these difference were not significance (*P*=0.992).

## Discussion

This study evaluated the tooth discoloration tendency of a nano-particle ZOE sealer in comparison with AH-26 and Pulp Canal sealer. It is important to make persistent efforts to manufacture a sealer that has all the ideal properties reported by Grossman [1]. NZOE sealer was recently manufactured in Mashhad Faculty of Dentistry through assistance from the Department of Physics, Faculty of Basic Sciences, Ferdowsi University of Mashhad. This sealer have shown suitable physical and antimicrobial properties [11-13] and the present study showed that its discoloration potential was comparable to AH-26 and Pulp Canal Sealer.

In the present study, in order to evaluate the effect of sealer on tooth discoloration, the sealers were placed in the pulp chambers of maxillary incisors. In a similar study, Zare Jahromi *et al.* [20] placed AH-26 and ZOE sealers in the pulp chambers of maxillary incisors in order to evaluate the discoloration caused by these sealers. But some other studies selected premolar teeth for evaluation of sealer discoloration [21-23]. 

Like almost all earlier studies on tooth discoloration caused by sealers [14, 16, 17, 24], apical access was used and the occlusal surface was kept intact, in order to prevent changes in optical properties of samples due to restorative materials, microleakage and tooth structure loss. In this study the smear layer was not removed similar to routine clinical set-up.

In the present study, tooth discoloration was evaluated by spectrophotometry using Easy Shade device. Overall, within the limitation of this study, NZOE sealer caused tooth discoloration comparable to the two other sealers. In a study by van der Burgt *et al.* [23], ZOE sealer resulted in significant discoloration of teeth after 7 days. They also reported that AH-26 resulted in severe grayish tooth discoloration. Parsons *et al. *[14] reported that AH-26 and Pulp Canal sealer resulted in significant discoloration of tooth crowns. They attributed this severe discoloration to the presence of silver ions. In the study by Gurel [25] the pulp canal sealer resulted in severe tooth discoloration. In addition, in a study by Partovi *et al.* [22], discoloration by ZOE sealer was more severe than that by AH-26 sealer at all time intervals. In the present study, NZOE sealer resulted in less discoloration than AH-26 sealer on days 1 and 30. In the study carried out by Zare Jahromi *et al.* [20] discoloration by ZOE sealer was less than AH-26 after 4 months. In the present study, too, a decreasing trend in discoloration was observed with the NZOE sealer, *i.e.* with the passage of time and after 3 days, discoloration began to decrease and was less than AH-26 after 30 days. However, there were no significant differences in discoloration caused by NZOE compared to AH-26 and Pulp Canal Sealer. 

Finally, in order to make an absolute conclusion, more evaluation on the discoloration potential of this new sealer must be done with a larger sample size and a longer time period. 

**Table 1.** Mean (SD) of *∆E* value of sealers in all experimental intervals

**Sealer (N) **	**1** ^st^ ** day ** ***∆E***_1_	**3** ^rd^ ** day ** ***∆E***_2_	**7** ^th^ ** day ** ***∆E***_3_	**1** ^st^ ** month ** ***∆E***_4_
**AH-26 (20)**	15.60 (14.33)	11.33 (9.69)	11.62 (9.08)	13.77 (16.01)
**Pulp Canal Sealer (20)**	9.99 (6.62)	9.99 (8.01)	12.11 (8.30)	10.50 (7.94)
**Nano Sealer (20)**	10.34 (6.00)	15.66 (11.23)	13.29 (7.66)	12.28 (7.32)

## Conclusion

Based on the results of the present study, it can be concluded that nano zinc oxide‒eugenol sealers cause similar tooth discoloration comparable to that caused by AH-26 and pulp canal sealers.
